# How to Improve Sustainability in Fused Filament Fabrication (3D Printing) Research?

**DOI:** 10.1002/gch2.202300408

**Published:** 2024-04-23

**Authors:** Cristiane Kalinke, Robert D. Crapnell, Paulo R. de Oliveira, Bruno C. Janegitz, Juliano A. Bonacin, Craig E. Banks

**Affiliations:** ^1^ Institute of Chemistry University of Campinas (UNICAMP) Campinas São Paulo 13083–859 Brazil; ^2^ Faculty of Science and Engineering Manchester Metropolitan University Chester Street Manchester M1 5GD UK; ^3^ Department of Nature Sciences Mathematics, and Education Federal University of São Carlos (UFSCar) Araras São Paulo 13600–970 Brazil

**Keywords:** 3D printing, circular economy, fused filament fabrication, recycling, sustainability

## Abstract

This review aims to provide an overview of sustainable approaches that can be incorporated into well‐known procedures for the development of materials, pre‐ and post‐treatments, modifications, and applications of 3D‐printed objects, especially for fused filament fabrication (FFF). Different examples of conductive and non‐conductive bespoke filaments using renewable biopolymers, bioplasticizers, and recycled materials are presented and discussed. The main final characteristics of the polymeric materials achieved according to the feedstock, preparation, extrusion, and treatments are also covered. In addition to recycling and remanufacturing, this review also explores other alternative approaches that can be adopted to enhance the sustainability of methods, aiming to produce efficient and environmentally friendly 3D printed products. Adjusting printing parameters and miniaturizing systems are also highlighted in this regard. All these recommended strategies are employed to minimize environmental damage, while also enabling the production of high‐quality, economical materials and 3D printed systems. These efforts align with the principles of Green Chemistry, Sustainable Development Goals (SDGs), 3Rs (Reduce, Reuse, Recycle), and Circular Economy concepts.

## Graphical abstract

This review provides an overview of sustainable approaches that can be incorporated into well‐known procedures for the development of materials, pre‐ and post‐treatments, modifications, adjustments, and applications of 3D‐printed objects, especially for fused filament fabrication (FFF). All these strategies can minimize environmental damage, while also enabling the production of high‐quality, economical materials, and 3D‐printed systems.

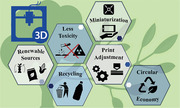

## 1 Overview

Additive manufacturing, or 3D printing, has emerged as an innovative technology for the manufacturing and design of new objects. This process involves layer‐by‐layer construction from digital models, enabling rapid prototyping to produce complex designs and shapes.^[^ ^1^ ^]^ Additive manufacturing has found applications in several fields, i.e., aerospace, healthcare, automotive, food, construction, energy, and sensors, among others, exploring thermoplastics, metals, concrete, and reinforced materials.^[^ ^1^, ^2^ ^]^ Due to accelerated advancement, the development of new materials, more efficient prototypes, and devices has also increased. On the other hand, environmental and health concerns have also emerged, due to the emission of toxic gases during the printing processes and the generation of waste.

In this scenario, the use of thermoplastic polymers in Fused Filament Fabrication (FFF raises plastic pollution as a huge issue. The production of plastics can lead to environmental problems during the industrial and post‐industrial processes, which involve the use of fossil fuels and toxic chemicals, deforestation, and environmental pollution. After use, plastic waste can be found in various locations, which include reuse or recycling, incineration, and disposal in landfills or the environment.^[^ ^3^ ^]^ The degradation of plastic in the environment is slow and depends on the plastic(polymer) type. Recently, microplastics have been highlighted as one of the biggest modern problems, polluting soil, air, and waters, mainly affecting aquatic ecosystems, and have also been found to accumulate in the human body.^[^ ^4^ ^]^ This is an alert for future problems of inappropriate plastic waste management. As an alternative, plastic waste can be easily reused or recycled and transformed into new feedstocks or final products. On the other hand, nowadays, less than 10% of plastic is recycled,^[^ ^5^ ^]^ which emphasizes this lack of concern with the global generation of waste.

Thus, the commitment to seek approaches aimed at decreasing, reusing, or recycling of plastic waste is essential. These practices allow waste to be reintegrated into the material cycle of production and use. From the perspective of FFF, the development of new sustainable materials and methods has stood out in industrial and scientific research, concentrating the attention on the use of less polluting feedstock materials and chemicals, recycling materials, and extending their useful life by circular economy (CE) applications.^[^ ^6^ ^]^ This can be achieved by manufacturing new materials from waste, old products in disuse, defective parts, prototypes, and printing supports that would otherwise be discarded. In this regard, a viable solution to be encouraged is the storage and post‐reuse of these materials that would eventually end up in landfills. Parallel to this, reinforcing filaments may be necessary once polymers lose their mechanical properties over time.^[^ ^7^ ^]^ For this purpose, the use of renewable or plant‐based materials would be ideal. Besides that, the search for greener methods and procedures that aim to minimize environmental impacts associated with the use of 3D printing technology is crucial.

These approaches are discussed in this review with a particular focus on FFF and applications, emphasizing the production of filaments from renewable sources (i.e., natural or plant‐based polymers, plasticizers, and reinforcements) and recycled materials (i.e., plastic waste, post‐industrial materials, and old parts). Additionally, alternative methods and procedures are also described to improve the sustainability of 3D printing, which includes adjusting printing parameters, and miniaturizing systems. Some applications of 3D‐printed devices and systems are also presented and discussed. Thus, this review aims to encourage the development of greener materials, procedures, and 3D‐printed devices.

## 2 Sustainable Development Initiatives

**Figure 1:**
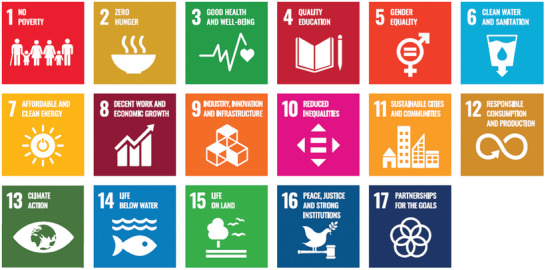
17 Sustainable Development Goals icons developed by the United Nations. Reproduced with permission from United Nations Department of Global Communications, Copyright (2023), United Nations.^[^ ^9^ ^]^ The content of this publication has not been approved by the United Nations and does not reflect the views of the United Nations or its officials or Member States.

A fundamental area of interest to the general public and wider scientific research is sustainability. This blanket term covers a wide range of initiatives that all essentially aim to achieve and maintain an ecological balance by avoiding the depletion of natural resources. One of the key global initiatives around this topic has been set up by the United Nations (UN), called the “Sustainable Development Goals”.^[^ ^8^ ^]^ These 17 goals were developed for the UN's 2030 Agenda for Sustainable Development and were adopted by all UN Member States in 2015 as an urgent call for action in a global partnership. These goals encompass a wide range of sustainable developments, aimed at the improvement of human lives globally, whilst also protecting the environment. They include aims such as “Goal **1** – No Poverty”, “Goal **2** – Zero Hunger”, “Goal **7** – Affordable and Clean Energy”, and of particular importance to the development of sustainable practices within additive manufacturing, “Goal **12** – Responsible Consumption and Production”, as shown in **Figure** **1**. This goal is focused on ensuring sustainable consumption and production patterns are implemented and maintained, with emphasis on developed countries taking the lead due to their 10 times larger environmental footprint. Among this UN goal are eight specific targets, such as 12.5 which states by 2030 that there should be substantially reduced waste generation through prevention, reduction, recycling, and reuse.

Strategies with similar approaches and goals have been released by numerous governing bodies worldwide. One of these examples includes the United Kingdom's recent “Plastic Pact”, linked to the Ellen MacArthur Foundation policies for a circular economy for plastic. Another program is the European Union's Plastics Strategy for Plastics in a Circular Economy. Both initiatives recognize the importance of plastic materials to our economies and daily lives; however, also acknowledge the serious negative effects they can have on the environment and human health. They suggest and are striving toward the elimination of problematic plastics, building stronger recycling systems, considering every stage of a product journey (before and after consumer use), and reaping the strong economic, social, and climate benefits of a transition to a functional circular economy.

**Figure 2:**
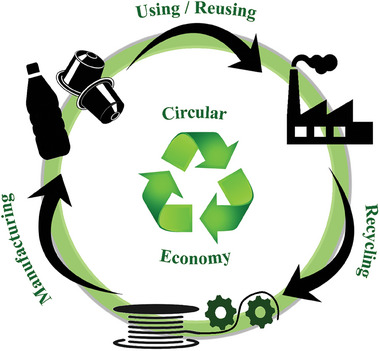
Illustrative scheme of the circular economy. Reproduced with permission from E. Sigley et al., *ACS Sustainable Chemistry & Engineering* 11, 2978−2988, Copyright (2023), ACS.^[^ ^11^ ^]^

The concept of a circular economy (CE) remains without a formal definition, however, there have been many operational definitions produced. Kirchherr and coworkers ^[^ ^10^ ^]^ found that the core normative idea in the conceptualization of a CE is that environmental sustainability, economic prosperity, and social equity are valid objectives. They took this further to produce a meta‐definition saying that “a circular economy is an economic system that replaces the “end‐of‐life” concept with reducing, alternatively reusing, recycling and recovering materials in production/distribution and consumption processes”.^[^ ^6^ ^]^ This aligns with the definition given by the Ellen MacArthur Foundation, which states that “the circular economy is a system where materials never become waste and nature is regenerated”, as illustrated in **Figure** **2**. It is important to observe that the circular economy term involves a multitude of steps or can even combine multiple cycles. For example, a waste product from one cycle, once not viable to re‐enter that cycle could become the feedstock for another. This clearly shows that the perfect circular economy cannot currently exist when it relates to additive manufacturing, due to the degradation of the polymeric material after being subjected to multiple thermal processing steps. Even so, the concept can be strived for, including the optimization of the smaller steps that form part of circular economies, particularly the “3Rs” of reducing, reusing, and recycling. Throughout this publication, we look to summarise the research taking place globally to help bring some of these concepts into additive manufacturing, whilst offering critical analysis of methodologies and offering insights into where we believe the next stages of improvements can be sought.

## 3 Sustainable Materials and Methods for FFF

The development of more efficient and environmentally friendly materials emerges every decade. This can be related to the increase in concerns about the use of petroleum‐derived materials, which, in addition to being finite resources, lead to concern regarding their environmental impact.^[^ ^12^ ^]^ Parallel to this, additive manufacturing technology has become a popular tool in scientific research, facilitating the manufacturing of general apparatus, tools, devices, models, and prototypes.^[^ ^13^ ^]^ In this context, FFF is probably the most used method for additive manufacturing, in which a thermoplastic filament is extruded, and layer‐by‐layer deposited on a table to obtain a new object.^[^ ^14^ ^]^ The type of material is probably the most important control parameter for FFF since it should improve surface and mechanical qualities.^[^ ^15^ ^]^ Therefore, it is important to choose feedstocks that meet these characteristics, allowing desirable malleability and printability, not losing their mechanical properties.

Furthermore, the toxicity of thermoplastics is still considered a major concern mainly due to the release of toxic gases during thermal processes, i.e., extrusion, 3D printing, and post‐treatments. For example, to compare the FFF method with stereolithography (SLA), researchers discovered that ABS parts printed by both showed some toxicity in fish embryos, although FFF presented significantly lower toxicity than SLA, proving to be a safer technique.^[^ ^16^ ^]^ As an alternative, a post‐treatment based on ultraviolet light was conducted on the SLA‐printed specimens, mitigating their toxicity. This exemplifies how researchers have made efforts to search for alternative, environmentally friendly materials and methods with similar or improved characteristics compared to conventional or commercial ones.

### 3.1 Production of Filaments from Renewable Sources

The search for greener feedstocks for the production of filaments has gone to the side of renewable source materials replacing conventional polymers, plasticizers, and other additives. **Table** **1** shows examples of sustainable bespoke filaments reported in the literature. In this instance, biopolymers, which can be either biodegradable or not, are successful examples of sustainable materials for additive manufacturing, especially for the FFF technique, which includes PLA (polylactic acid), PHA (polyhydroxyalkanoates), PVA (polyvinyl alcohol), PA‐11 (polyamide 11 or nylon 11), and others.

**Table 1:** Sustainable reinforced filaments for FFF additive manufacturing.

Base polymer	Filler / Blend	Pretreatment	FFF additive manufacturing	Considerations	Reference
61.6 wt.% coffee pods recycled PLA	29.6 wt.% CB, 8.8 wt.% PES	Alkali (0.5 mol L^−1^ NaOH) and electrochemical	Electrochemical devices at 215 °C, and 60 mm s^−1^ print speed	Flexible filament. Enhanced conductivity and electrochemical properties for caffeine detection. Non‐conductive filament recycled three times.	[11]
65 wt.% coffee pods recycled PLA	25 wt.% CB, 10 wt.% castor oil	Alkali (0.5 mol L^−1^ NaOH) and electrochemical	Electrochemical devices at 225 °C, and 40 mm s^−1^	Flexible filament. Enhanced electrochemical performance for BPA detection.	[20]
95 wt.% PLA	5 wt.% lignin	−	Testing specimens at 205 °C	The protect layer avoids the diffusion of oxygen. The filament still brittle.	[22a]
70 wt.% PLA/PHA	30 wt.% pine wood fiber	−	Testing specimens at 220 °C, 23 mm s^−1^	The elongation at break remains after 3DP.	[22b]
95 wt.% PLA	5 wt.% walnut shell	Alkali (NaOH) for 3 h and silane for 8 h	Testing specimens at 210 °C, and 50 mm s^−1^	Improvement in the mobility of filament due to the walnut shell lubricant effect.	[22d]
60 wt.% PLA	26.3 wt.% potato starch, 13.3 wt.% plant glycerin, 0.4 wt.% epoxydized soybean oil	−	Anatomical and porous models at 185 °C, and 20−80 mm s^−1^	Improvement of hydrophilicity, degradation, and compostability.	[22e]
75 wt.% ABS	25 wt.% basalt fiber	−	Testing specimens at 250 °C, and 30 mm s^−1^	Improvement in tensile and flexural strength.	[23]
92.7−100 wt.% PA‐11	0−7.3 wt.% sepiolite nanoclay	−	Testing specimens at 280 °C, and 10 mm s^−1^	Enhanced mechanical strength elastic modulus, and dimensional accuracy.	[24]
30 wt.% virgin, 70 wt.% waste and PPE recycled PLA	−	−	−	Similar quality for recycled and virgin PLA filaments.	[3]
91.1 vol% recycled PLA	8.9−10 vol% recycled carbon fiber	−	Testing specimens at 210−240 °C, and 100−200 mm s^−1^	Improved tensile and bending strength for the recycled PLA and carbon fiber filament.	[30]
65 wt.% coffee pods recycled PLA	15 wt.% CB, 10 wt.% MWCNT, 10 wt.% PES	Alkali (0.5 mol L^−1^ NaOH) for 30 min	Electrochemical biodevices at 215 °C, and 70 mm s^−1^	Flexible filament. Enhanced bioreceptor binding and electrochemical performance for yellow fever virus detection.	[31a]
75 or 100 wt.% 3DP old devices recycled PLA	25 wt.% CB	Alkali (0.5 mol L^−1^ NaOH) and electrochemical	Electrochemical devices at 210 °C and 70 mm s^−1^ or 202 °C and 35 mm s^−1^ (PLA or CB/PLA filaments)	Enhanced electrochemical performance for acetaminophen detection. Filament recycled twice.	[31b]
95−99.5 wt.% bottles recycled PET	0.5−5 wt.% packaging waste biochar	−	Testing specimens at 270 °C, and 50 mm s^−1^	Improvement in mechanical and thermal properties.	[32]
65 wt.% coffee pods recycled PLA	15 wt.% CB, 10 wt.% graphite, 10 wt.% castor oil	Electrochemical	Electrochemical sensor at 215 °C, and 70 mm s^−1^	Flexible filament. Enhanced electrochemical performance for oxalate.	[34]
Broken 3DP parts recycled PLA	Bioinspired polydopamine	−	Testing specimens at 210 °C	Improvement in tensile strength and strain at break for polydopamine coated PLA.	[35]
Meal bags recycled LLDPE/LDPE polymers	−	−	Testing specimens, water vapour barriers, and medical finger cots at 285 °C, and 4 mm s^−1^	Mechanical and barrier properties comparable to the pristine material.	[36]
70−100 wt.% recycled nylon‐6	5 wt.% ABS or 30 wt.% TiO_2_	−	Testing specimens at 230−235 °C, and 40−50 mm s^−1^	Enhanced mechanical properties, thermal stability, and warping effects for the non‐filled filament.	[37]
70 wt.% ABS	30 wt.% starch, 1 wt.% SMA, 2 wt.% MBS, 5 wt.% TiO_2_ or CB	−	Different specimens at 210 °C	Improved mechanical, thermal resistance, flowability properties, and low emission of VOC.	[38]

**Note**: 3DP: 3D‐printed; ABS: Acrylonitrile‐butadiene‐styrene; BPA: Bisphenol‐A; CB: Carbon black; LDPE: Low‐density polyethylene; LLDPE: Linear low‐density polyethylene; MBS: Methyl‐methacrylate butadiene styrene; MWCNT: Multi‐walled carbon nanotubes; PA‐11: Polyamide‐11; PES: Poly(ether‐sulfone); PET: Polyethylene terephthalate; PHA: Polyhydroxyalkanoate; PLA: Polylactic acid; PPE: Personal protective equipment; SMA: Styrene‐maleic anhydride copolymer; VOCs: Volatile organic compounds.

In this instance, biopolymers produced from renewable sources, such as sugars and starches, can be easily biodegradable and compostable.^[^ ^17^ ^]^ Considering this, PLA is probably the most attractive thermoplastic polymer due to its bio‐based, biodegradable, and biocompatible characteristics.^[^ ^18^ ^]^ On the other hand, bio‐based non‐biodegradable polymers (i.e., bio‐PET, bio‐PE, and bio‐PA) are also found and can be recycled as well.^[^ ^19^ ^]^

In addition, to the use of alternative base polymers, plasticizers have been studied as additives to improve the flexibility and printability of the filaments.^[^ ^11^, ^20^ ^]^ This has avoided the loss of mechanical and thermal properties due to the breaking of the polymer chains and/or its degradation, which is commonly observed for biopolymers. The produced materials have shown excellent malleability even in filled filaments, demonstrating the success of this modification.

**Figure 3:**
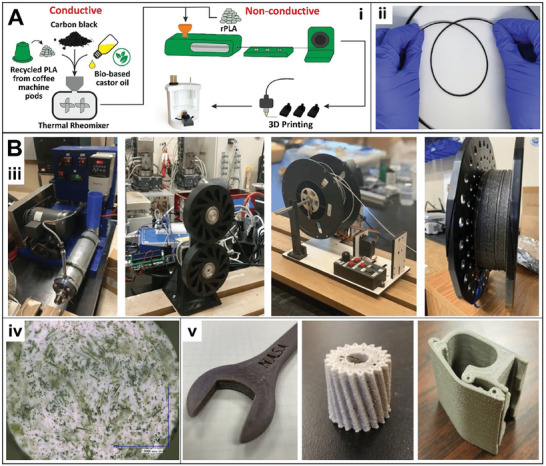
A) Extrusion process for fabrication of flexible conductive and non‐conductive filaments based on recycled PLA and castor oil bioplasticiser i‐ii). B) ABS and basalt fiber reinforced filament: Extrusion system iii), cross section image iv), and 3D printed objects (open‐faced wrench, gear, and clamp) v). Reproduced with permissions from R. D. Crapnell et al., *Green Chemistry*, 25, 5591–5600, Copyright (2023), RSC ^[^ ^20^ ^]^ (A); and N. Coughlin et al., *Journal of Composites Science*, 3, 89, Copyright (2019), MDPI ^[^ ^23^ ^]^ (B).

Vegetable oils are renewable options obtained from plants through mechanical pressing or solvent extraction. Oils can act as lubricants, reducing intermolecular friction between polymer chains, facilitating their movement, increasing the distance between polymer chains, decreasing the glass transition temperature of the polymer, and turning it more malleable.^[^ ^21^ ^]^ As an example, the use of castor oil as a bioplasticiser for the development of conductive and non‐conductive filaments is reported.^[^ ^20^ ^]^ Castor oil is extracted from castor bins Ricinus communis plant, also known as ricin oil. 10 wt.% oil was sufficient to produce a filament with excellent flexibility and electrical conductivity (**Figure** **3**A). Also, the castor oil filament showed similar thermal stability compared to a recycled PLA filament without oil as plasticiser, demonstrating that the addition of oil has no disadvantages on the thermal characteristics of the produced filaments.

The reinforcement of base polymers is also described by using other plant‐based materials (i.e., lignin, wood, fibers, shells, starches, etc), mainly for the improvement in mechanical and thermal properties.^[^ ^22^ ^]^ For example, lignin can improve some negative aspects of PLA in filaments, such as low thermal stability and high degradation since it works as a protection layer avoiding the diffusion of oxygen.^[^ ^22a^ ^]^ In another work, basalt fiber was used for the reinforcement of ABS‐based filament, aiming at the construction of 3D printed objects using basalt extracted on Mars.^[^ ^23^ ^]^ The mixed material showed a homogeneous distribution of basalt fibers of an average size of 280 µm in length. Different 3D‐printed objects proved to be more rigid than using only ABS (Figure 3B). In addition, experiments conducted at the International Space Station demonstrated that objects can be manufactured in microgravity using ABS base polymer.

**Figure 4:**
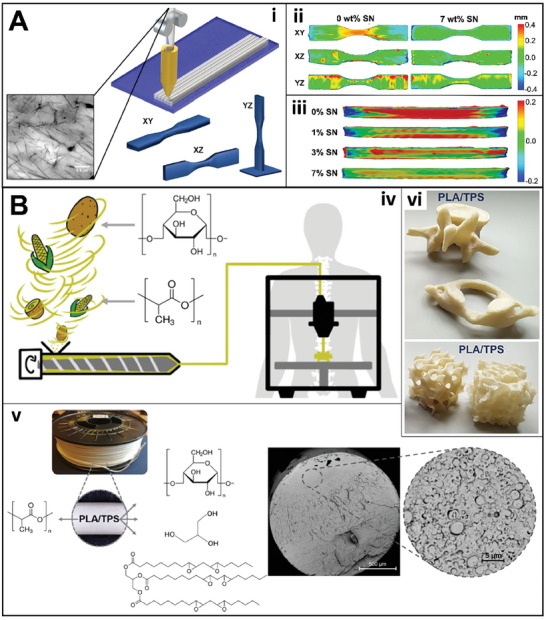
A) Polyamide 11 and sepiolite nanoclay (SN) reinforced filament: Homogeneous distribution of SN into the filament obtained by TEM i); Testing specimens heat maps according to the 3DP orientation ii), and the SN proportion iii). B) PLA, potato starch, plant glycerol, and epoxidized soybean oil biodegradable and compostable filament for 3DP iv): Cross‐section SEM image v), and 3DP vertebrae and porous models vi). Reproduced with permissions from M. Herrero et al., *ACS Sustainable Chem. Eng*. 6, 12393−12402, Copyright (2018), ACS ^[^ ^24^ ^]^ (A); and A. Haryńska et al., *ACS Sustainable Chem. Eng*. 9, 6923−6938, Copyright (2021), ACS ^[^ ^22e^ ^]^ B).

In another approach, sepiolite nanoclay was incorporated into polyamide‐11 biopolymer, in which the elastic modulus is correlated to the printing orientation and the proportion of the nanoclay.^[^ ^24^ ^]^ In this case, 3DP specimens obtained by the end‐on (YZ) printing orientation and higher filler proportion (7 wt.%) showed less warping deformation, as observed by the heat maps (**Figure** **4**A). Thus, it is evident that the reinforcement with the nanoclay led to the improvement of the mechanical properties of the filament. The blend of different renewable materials is also reported in the literature. For example, potato starch is a highly biodegradable and abundant biopolymer; while soybean oil is commonly used as a plasticizer, improving the brittleness and low flexibility of polymers. Thus, the combination of properties of both materials plus PLA and plant glycerol was used to produce a filament with improved hydrophilicity, hydrolytic degradation, and compostability.^[^ ^22^, ^25^ ^]^ This demonstrates the innovative aspects related to the production of eco‐friendly biopolymers. Also, the filament was used to obtain 3DP complex anatomical models and porous structures with high printing quality (Figure 4B).

### 3.2 Production of Filaments from Recycled Sources

**Figure 5:**
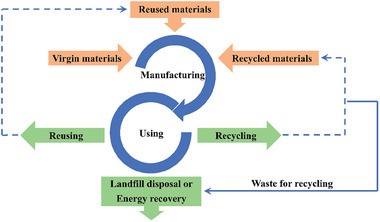
Reuse and recycling of materials for additive manufacturing. Reproduced with permissions from G. Muñoz et al., *Applied Sciences*, 10 (24), 8967, Copyright (2020), MDPI.^[^ ^3^ ^]^

Although thermoplastic polymers are widely used as materials for FFF in various industrial and research areas, they represent the majority of plastic waste currently generated, ≈78%.^[^ ^26^ ^]^ Alternatively, thermoplastics can be reused or recycled in different ways, as exemplified by **Figure** **5**, minimizing the plastic waste in the environment or landfills.^[^ ^3^ ^]^ Reuse is one of the simplest strategies to repurpose plastic waste; however, in the future, this plastic will become waste. Thus, plastic waste has to be considered as a resource to be reintroduced to the plastic life cycle, closing the circular economy looping.^[^ ^27^ ^]^ This has attracted the attention of researchers to produce new materials from recycled sources, for example, to make bespoke filaments for FFF. It is important to highlight that sustainable waste recovery, treatment, and recycling can also be considered a challenge to minimize environmental pollution; thus, different physical, chemical, or biological methods can be used. To produce filaments, re‐extrusion has been the widely used method since it permits the manufacturing of new objects and products with similar performance compared to new filaments.^[^ ^26^ ^]^

Recycling involves a process of degradation of the material, causing the break of polymer chains. At this point, recycling or reprocessing can affect the mechanical and thermal properties of the polymer, such as mechanical and impact strength, melt flow index, transition and degradation temperatures, molecular weight, and others.^[^ ^28^ ^]^ Therefore, knowledge about the changes and implications caused by the recycling of each thermoplastic is essential and allows the user to apply the recycled material to the most efficient use. For example, to create adequate filament for FFF the polymer must be of a higher quality than what is necessarily required for other potential applications such as injection molding. Sigley and co‐workers ^[^ ^11^ ^]^ found that they could re‐extrude recycled PLA filament up to three times and still produce quality filament. Further research needs to be done in these cases as there is no reason the waste from this final filament could not be used alongside virgin PLA to make new filament or used within other applications.^[^ ^29^ ^]^ The recycling and remanufacturing of filaments have been reported for the production of new filaments.^[^ ^30^ ^]^ High recoveries have been achieved for carbon fiber (100%) and PLA (73%) after recycling and remanufacturing new filaments, demonstrating the outstanding success of the proposed method. In addition, recycled filaments have improved mechanical properties, i.e., tensile and bending strength. In this case, the energy consumption for recycling and remanufacturing processes can be considered an issue to be overcome, reducing energy consumed to achieve greener methods.

**Figure 6:**
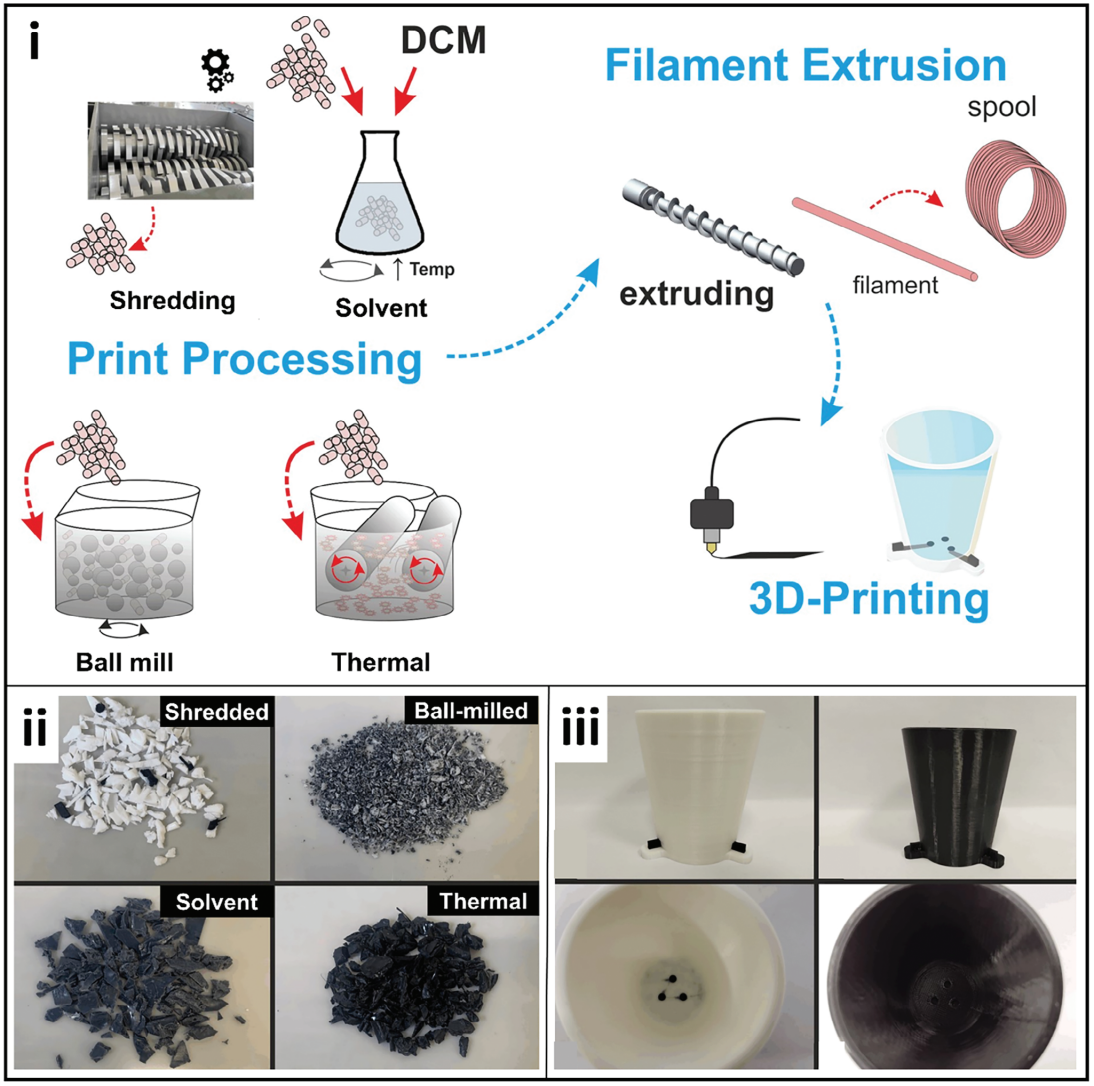
Recycling processes (shredding, solvent, ball mill, or thermal mixing) for the fabrication of filaments based on PLA recycled from old electrochemical cells i): Mixed materials obtained from different processes ii), and 3D printed electrochemical cells from original (white cell) and recycled (black cell) PLA‐based filaments. Reproduced with permissions from R. D. Crapnell et al., *ACS Sustainable Chemistry & Engineering* 11 (24), 9183–9193, Copyright (2023), ACS.^[^ ^31b^ ^]^

Post‐industrial materials have also been recycled and re‐extruded for the manufacturing of PLA‐based filaments, as reported, by using coffee machine pod waste and old electrochemical cells.^[^ ^11^, ^20^, ^31^ ^]^ In this case, PLA‐based waste was processed and transformed into pellets for direct extrusion aiming at the fabrication of non‐conductive filaments. The incorporation of carbon‐based fillers (i.e., carbon black and/or carbon nanotubes) and plasticizers can be easily performed by thermal mixing since a homogeneous and high‐quality filament is obtained compared to other methods, such as granulation, ball‐milling, and solvent mixing,^[^ ^31b^ ^]^ as shown in **Figure** **6**. After this, the method also included the granulation of the mixing material and its extrusion to obtain the conductive filaments. Recycled PET has also been used for this purpose by the recycling of postconsumer PET bottles for the production of PET filament.^[^ ^32^ ^]^ In this case, the filament was reinforced with a graphitized biochar produced from packaging waste. This demonstrates the production of a fully recycled filament with improved mechanical, thermal, and dynamic properties.

In addition, the recycling process can be performed for limited cycles, without adding virgin materials or additives, before the materials lose mechanical properties, which is essential to guarantee the quality of the 3D printed specimens. This can be associated with the polymer type, the recycling, and the manufacturing processes. After some cycles of recycling and remanufacturing, induced aging processes and degradation of the polymer are pointed out as the causes for changes in the mechanical performance, affecting the printability and quality of the filaments.^[^ ^33^ ^]^ Recently, the manufacturing of electrochemical devices by recycling old devices based on PLA was reported, showing that thermal recycling could be performed three times without printing failures.^[^ ^11^ ^]^ PLA recycled filaments were also fabricated from waste, old visors, and personal protective equipment parts used during the COVID‐19 pandemic.^[^ ^3^ ^]^ In this work, the Material Circularity Indicator (MCI) of the produced material was calculated showing a high level of circularity, and efficiency in the recycling process.

This represents a synergism between the production of new materials and the circular economy concept, allowing the maintenance of the useful life of a material. Hence, scientific research into the production of recycled and renewable materials holds significance not only in the context of the environmental and economic point of view, but it also allows researchers understand the freshness and challenges associated with the development of eco‐friendly materials and procedures. Further work in this area should be focussed on identifying key physicochemical and mechanical data in polymer sources that can be used to find the best use. Most of the examples discussed look to recycle filament or prints back into filament, even though this may not be the most efficient use of that material. Identifying the properties of waste polymer sources can be the key to application identification in the future.

### 3.3 Reduction or Use of Less Toxic Chemicals

Some processes related to the development of new materials, such as filaments, involve the use of chemicals for the production, modification, and activation processes. In this instance, the reduction of reagent use, or the application of less toxic chemicals can be considered to achieve more sustainable methods and materials. As seen before, several renewable materials have already been studied in substitution of conventional chemicals. Furthermore, carbon‐based conductive fillers (i.e., graphite, carbon black, and graphene, among others) have been widely employed in the replacement of potentially hazardous materials, such as some metals.^[^ ^39^ ^]^ The exchange, reduction, or lower consumption of toxic chemicals also reflects on safer characteristics of the generated waste, facilitating its treatment and disposal. In this case, the waste toxicity can be reduced by treatments such as recycling, degradation, and passivation.^[^ ^40^ ^]^

For the development of conductive filaments, the use of additional activation treatments is normally required to improve their performance. These processes can be carried out in the presence of organic solvents, buffered, acid, or basic solutions, in which the isolating polymer can be removed on the surface of the material, by dissolution or saponification reactions, exposing the conductive material and improving the electrical conductivity.^[^ ^41^ ^]^ Nevertheless, a greener alternative is not using solvents, which could be a successful substitute for electrochemical, mechanical, and laser activation methods.

The electrochemical activation has been performed by the application of potential in the presence of phosphate buffer or sodium hydroxide solutions. This leads to the oxidation or reduction of the material surface, which is an advantage for the development of electrochemical sensors, for example, allowing the improvement of active interaction sites.^[^ ^11^, ^42^ ^]^ The mechanical activation by polishing 3D printed surfaces based on filaments using an abrasive paper is reported, generating a high surface area, and smoother characteristics. In addition, the removal of the surface polymer promotes the exposure of the conductive material allowing the increase of the conductivity in electrochemical devices.^[^ ^43^ ^]^

Similarly, laser activation is a clear post‐processing method, in which polishing is performed with a CO_2_ laser.^[^ ^44^ ^]^ The laser acts by breaking surface polymeric chains, and the melted material flows filling valleys and removing defects on the polymeric surface. As a result, the improvement in mechanical properties (i.e., tensile and flexural strength) and surface roughness can be achieved.^[^ ^45^ ^]^ In addition, laser post‐treatment shows no toxic effects, which is ideal for medical applications.^[^ ^46^ ^]^ Although side effects can occur, such as material discoloration, and the formation of bubbles, the laser parameters should be optimized according to the type of material, which includes the laser power, temperature, and interaction time.^[^ ^47^ ^]^ Thus, polishing surface treatments can be highlighted due to their remarkable biocompatible and eco‐friendly advantages compared to other methods, which include no chemicals or solvents used, less processing time, and no waste generated.^[^ ^44^ ^]^

### 3.4 Adjustment of Printing Parameters

The printability and quality of 3D‐printed objects can also be enhanced through adjusting or optimizing printing parameters.^[^ ^48^ ^]^ Nozzle and bed temperatures, printing speed, orientation, layer thickness, and infill density are some of the printing parameters that can be evaluated. For instance, temperature significantly influences the mechanical resistance of the final object. Low temperatures can affect the layers' adhesion and may generate a lack in the printed object.^[^ ^49^ ^]^ The blockage of the nozzle can also occur since the temperature is insufficient to properly melt and extrude the material. On the other hand, excessive temperatures can result in a melted appearance of the filament, spending more material, and affecting the layers appearance.^[^ ^39b^ ^]^ Hence, considering the used material and its melting point is crucial when adjusting temperature settings. For example, PLA has a glass transition temperature of 70 °C and a melting temperature of 150 °C,^[^ ^49^ ^]^ which must be taken into account for extrusion and printing processes. Recommended temperatures for PLA typically 190–220 °C for the extruder and 35–60 °C for the bed are employed.^[^ ^39b^ ^]^ Therefore, it is essential to follow the manufacturers' recommendations and understand the properties of each material, to establish appropriate parameters and achieve high printing quality.

Choosing inadequate printing parameters typically results in extended printing times and increased material and energy wastage. Moreover, the printed object or device may be unusable due to failures and defects. To mitigate these issues, sufficient internal infill density is essential to support all layers without failure.^[^ ^50^ ^]^ Additionally, evaluating layer thickness is crucial, as it determines the number of layers required for printing an object. For example, lower layer thickness necessitates a higher number of layers. Also, these parameters directly impact the resolution, accuracy, strength, and electrochemical performance of 3D‐printed specimens.^[^ ^39^, ^51^ ^]^ Therefore, utilizing a low layer thickness results in a high resolution, as well as increased tensile and flexural strengths.^[^ ^52^ ^]^

Thus, according to various parameters that can be altered, temperature, printing speed, infill density, and layer thickness, have been highlighted since they directly affect energy and material consumption, costs, time of printing, and waste generation.^[^ ^45^, ^50^, ^53^ ^]^ Considering the utilization of FFF technology, it is recommended to evaluate printing parameters collectively to strike a balance between the sustainability and print quality of the final 3D printed parts. Furthermore, the choice of methods, chemicals, and other materials employed in FFF significantly impacts the environmental footprint of 3D printing technology.

## 4 Miniaturization of Systems

Another strategy consists of reducing the size of 3D printed devices and systems, and obtaining miniaturized devices, which leads to a reduction in the amount of construction material required and also in the amount of material or inputs in its application. Although the development of miniaturized devices in analytical chemistry can be traced back to the late 20th century, this concept of miniaturization and integration of multiple analytical functions onto a single platform has been renewed and incorporated into 3D printed systems.^[^ ^54^ ^]^ The most important strategies consist of capillary electrophoresis,^[^ ^55^ ^]^ lab‐on‐a‐chip devices,^[^ ^56^ ^]^ microfluidics and micrototal analysis systems (µTAS),^[^ ^57^ ^]^ and point‐of‐care (POC) diagnostics,^[^ ^58^ ^]^ among others.^[^ ^59^ ^]^ Based on this, the use of miniaturized systems offers several advantages, such as an increase in analytical frequency, enhanced sensitivity, portability and *in loco* applications, integration of multiple functions, low‐cost, and versatility. In addition, the reduction of environmental impact is also reported, by reducing sample and reagent consumption, and generated waste.^[^ ^54^, ^60^ ^]^ On the other hand, miniaturized systems also show some challenges, such as fabrication complexities and potential issues related to scaling down certain processes. However, exploring new materials, fabrication methods, and applications allows the enhancement of the capabilities of miniaturized and portable systems for overcoming analytical challenges.

Several techniques can be employed to create and enhance miniaturized systems, depending on the application and applied material. Some of the most used techniques include microfabrication by photolithography,^[^ ^61^ ^]^ micro‐electro‐mechanical systems (MEMS),^[^ ^62^ ^]^ bulk micromachining,^[^ ^63^ ^]^ nanoimprint lithography,^[^ ^64^ ^]^ bottom‐up nanofabrication,^[^ ^65^ ^]^ soft lithography,^[^ ^66^ ^]^ and additive manufacturing.^[^ ^67^ ^]^ Regarding 3D printing, the obtaining of microdevices is possible by building pieces or small structures with high precision in terms of form and size.^[^ ^68^ ^]^ 3D printing exhibits highly attractive characteristics, primarily stemming from its FFF approach, which leads to minimal waste generation during the manufacturing process or analysis, using these devices.^[^ ^69^ ^]^

## 5 Conclusions and Perspectives

Sustainable alternatives can be highlighted for FFF. The use of renewable and/or recycled materials has allowed various advantages, mainly the availability of raw materials with reduced environmental impact, and the reduction in production costs. Parallel to this, the recycling of materials, especially plastic, permits the reintroduction of waste into the life cycle of materials, aiming for a circular economy of materials, increasing their usefulness, and avoiding their disposal. In addition, the production of bespoke materials led to an improvement in the performance of 3D‐printed specimens, allowing the modulation of thermal, mechanical, and conductive properties in several application areas. Alternative solvent‐free methods are also greener possibilities that should be incorporated into pre‐ and post‐treatment processes, for example, by using mechanical, electrochemical, and laser methods. Furthermore, the adjustment and optimization of the FFF parameters have demonstrated advantages, contributing to the reduction of energy consumption, costs, printing time, and waste generation. The miniaturization of systems is another covered approach, which allows the use of less material, reducing production costs, and facilitating their portability. All these aspects demonstrate that new, high‐quality materials can be ecologically and economically produced, modified, and applied in several academic and industrial areas. Finally, we would like to highlight that discussions about greener practices are urgent and deserve to be encouraged not only in research laboratories, but also in other public and private sectors since this is a global concern.

## Conflict of Interest

The authors declare no conflict of interest.
